# Characteristics of Serve, Reception and Set That Determine the Setting Efficacy in Men’s Volleyball

**DOI:** 10.3389/fpsyg.2020.00222

**Published:** 2020-02-18

**Authors:** Jara González-Silva, Carmen Fernández-Echeverría, Manuel Conejero, M. Perla Moreno

**Affiliations:** ^1^Didactic and Behavioural Analysis in Sports Research Group, Faculty of Sport Sciences, University of Extremadura, Cáceres, Spain; ^2^Faculty of Education Sciences, University of Seville, Seville, Spain; ^3^Faculty of Sport, University of Granada, Granada, Spain

**Keywords:** performance, volleyball, set, high level, male

## Abstract

The aim of this investigation was to establish the criteria of service, reception and set that determine setting efficacy in world-class top-level volleyball. The study sample consisted of 4.113 gaming actions (1.371 serve actions, 1.371 reception actions, and 1.371 set actions), corresponding to the observation of four matches for each of the 12 best ranked teams in the Volleyball World Championship - a total of 23 matches. The criteria were: in-game role of the server, serve zone, type of serve, striking technique and serve direction; receiver player, reception zone, and reception efficacy; setting zone, type of set, setting technique, setting efficacy, a set’s area, and set tempo. Multinomial logistic regression showed that criteria related to reception (reception efficacy) and to set (setting zone, type of set, a set’s area, and set tempo) determined set efficacy. Specifically, positive and negative receptions and settings from acceptable and non-acceptable zones reduced perfect setting. In contrast, the jump set toward zones three and six and the first and second tempo increased perfect setting. Serve criteria did not determine set efficacy. This study can guide trainers and players in the training process.

## Introduction

The specific characteristics of volleyball imply that its game actions are interrelated. Except for the serve, each action is influenced by a preceding action, and all actions influence subsequent actions (Eom and Schutz, 1992). The serve, attack, and block actions have the highest correlation with victory (Marcelino et al., 2008; Montoro-Escaño and Hernández-Mendo, 2014), as they correspond to terminal actions that allow directly scoring points (Marcelino et al., 2010). Reception, set, and defence are intermediary linking actions that do not usually allow obtaining direct points (Marcelino et al., 2010). Teams lacking the ability to effectively execute such intermediate actions are usually most likely to lose sets (Silva et al., 2014).

In volleyball, many studies have been carried out on the research topic of match analysis. Most investigations involved the finalist actions, serve (Buscá et al., 2012), attack (Marcelino et al., 2012; Costa et al., 2017), or block (Afonso and Mesquita, 2011), with fewer studies of the intermediate actions, reception (Paulo et al., 2016), set (Silva et al., 2014), and defence (Mesquita et al., 2007). These investigations have focussed on determining general aspects of the game through descriptive analysis (Callejón and Hernández, 2009) or specific information of the performance of main aspects through inferential analysis (Ureña et al., 2002; Afonso et al., 2005a, b), analysing the variables that could predict performance (González-Silva et al., 2016).

At present, players’ performance variability and the non-linear changes of game actions are important elements to understand sports dynamics (Hamil et al., 1999; Oullier et al., 2006). Thus, studies are developed through entropy, which has allowed researchers to determine players’ performance variability (Rhea et al., 2011; Ramos et al., 2017). Due to the sequential nature of volleyball, knowledge of the relationships between the different game actions is essential. The analysis of the interaction between game actions through social networks provides information about the relationships between the different elements of the system through the establishment of a network system (Laporta et al., 2018).

However, few studies have actually investigated the relationships between the different actions (Costa et al., 2011), and there is an urgent need for studies going beyond the analysis of single actions. The dynamic nature of volleyball makes implies taking the relationships between game actions into account (Hale, 2001).

Of the few studies that have investigated relationships between different actions, several have shown that serve technique greatly influences the efficacy of serve-reception (Joao and Pires, 2015; García-de-Alcaraz et al., 2016). Specifically, jump serves were found to increase the number of receptions that did not facilitate an attack on the opponent. The characteristics of the serve influenced its reception (Paulo et al., 2016). Research has also shown an influence of the serve on setting. At least one study has shown that the type of serve can influence the setting zone (Afonso et al., 2012). Powerful jump serves were more likely to be preceded by sets from acceptable zones. Other studies have shown that, before perfect receptions, setters were more likely to have executed setting of the first tempo, thus increasing the probability of gaining points (Bergeles and Nikolaidou, 2011), although Papadimitriou et al. (2004) failed to verify this finding. However, these authors did show that the quality of the reception influenced the setter’s offensive strategy. At high levels of volleyball, set efficacy was found to increase when using the finger set technique (Palao et al., 2009), thus indicating that set variables also influence setting efficacy. González-Silva et al. (2016) showed that, in the male category of training stages, setting zone, setting technique, a set’s area, and set tempo also influenced setting efficacy.

Previous investigations focussed on the training stages of volleyball have shown that setters usually did not perform a perfect setting action following a poor serve-reception (González-Silva et al., 2016). Conversely, for high-level volleyball, research has shown that the setter is often able to achieve success from bad serve-receptions (Papadimitriou et al., 2004; Silva et al., 2014). In addition to verifying this fact, the present investigation sought to determine whether variables related to service, receptions, and the setting action itself influenced the setting efficacy. Therefore, all actions prior to the setting action (a finalist and other intermediaries) are considered, an aspect that has not been analysed in prior studies.

## Materials and Methods

### Design

The present investigation is an ideographic, punctual and multidimensional observational design (Anguera et al., 2011).

### Participants

The study sample comprised a total of 4113 game actions (1371 serve actions, 1371 reception actions, and 1371 setting actions) corresponding to the observation of the 12 best classified teams in the men’s World Championship. The observed actions occurred in the third phase of the championship. All the matches of that phase were analysed, which involved the observation of four matches of each of the participating teams, that is, 87 sets, in which the two teams that played each of the matches were observed. The sets per team are shown in Table 1. Participants’ informed and written consent was obtained for the study.

**TABLE 1:** Number of sets observed by team.

**Team**	**Sets**	**Team**	**Sets**
Brazil	15	Bulgaria	16
Cuba	18	Germany	12
Serbia	13	Argentina	11
Italy	16	Czech Republic	15
Russia	14	France	14
EEUU	13	Spain	17

The study is exempt from ethical approval because the observation of game actions does not pose any risk to the participants. The study was performed in accordance with Spanish and international guidelines for scientific research involving humans.

### Instrument

The data collected were register with the observational analysis software applied to volleyball VA-Sports (Morante, 2014).

### Procedure

All the matches were recorded in their entirety, with the camera located in one of the corners of the court, guaranteeing an optimum field of vision.

After collecting the video footage, an observer, who was a Graduate of Science in Physical Activity and Sports, National Level III volleyball coach, and who had 5 years of experience as a coach, conducted a training process and encoded game actions. The training process was carried out for six training sessions using samples with different characteristics.

The intra-observer Cohen’s Kappa values reached in the observation of all the criteria were higher than 0.75, which was the minimum value considered to be almost perfect agreement (Fleiss et al., 2003). To guarantee the temporal reliability of the measurement, the same coding was performed on two occasions, with an interval of 10 days, obtaining Cohen’s Kappa values of over 0.75. Table 2 shows the Cohen Kappa values obtained in each training session of each criterion, at the different temporary moments.

**TABLE 2:** Kappa de Cohen values of the criteria in each training session.

**Criteria**	**1° training**	**2° training**	**3° training**	**4° training**	**5° training**	**6° training**	**Temporary**
In-game role of the server	0.902	0.990	0.990	0.990	0.990	0.990	0.990
Serve zone	0.688	0.688	0.680	0.790	0.851	0.884	0.885
Serve type	0.900	0.980	0.980	0.990	0.990	0.990	0.989
Striking technique	0.713	0.733	0.733	0.884	0.789	0.849	0.860
Serve direction	0.710	0.720	0.780	0.800	0.867	0.880	0.885
Serve efficacy	0.875	0.921	0.421	0.521	0.789	0.790	0.800
Receiver player	0.910	0.980	0.990	0.990	0.990	0.950	0.985
Reception zone	0.670	0.670	0.740	0.785	0.788	0.920	0.920
Reception efficacy	0.897	0.870	0.799	0.805	0.805	0.810	0.880
Setting zone	0.688	0.688	0.792	0.825	0.890	0.930	0.935
Type of set	0.930	0.990	0.990	0.990	0.990	0.990	0.990
Setting technique	0.950	0.990	0.990	0.990	0.990	0.990	0.990
Setting efficacy	0.834	0.798	0.840	0.849	0.880	0.900	0.900
Set’s area	0.990	0.990	0.990	0.890	0.930	0.990	0.990
Set tempo	0.759	0.759	0.759	0.756	0.825	0.881	0.880

Finally, a generalisability analysis (TG) has been carried out (Cronbach et al., 1963, 1972). This type of analysis has been used in order to know the validity of the sample. This analysis was carried out with SAGT, which is a computer application for generalisability analysis (Hernández-Mendo et al., 2016). For the study of validity, a three-faceted design was structured: coincidence (P), criterion (V), and category (C), which allow estimating the validity of the criteria used in the category system.

After obtaining the optimum reliability values, the observation was carried out. Below we indicate the criteria and their corresponding categories (Hernández Mendo et al., 2012; Anguera and Hernández-Mendo, 2013), considered in the observation tool used in this study:

In-game role of the server, defined as the in-game role of the player serving. The categories were: receiver, setter, opposite, and middle attacker (Araújo et al., 2010; Stankoviae et al., 2018).

Serve zone, defined as the zone from which the serve is carried out, covering a 9-m wide space located behind the baseline of the court and as an extension to the sidelines of the court, differentiating three zones of origin. The categories were: zone 1, defined as the strip 3 m wide from the right sideline of the field and behind the bottom line; zone 6, defined as the strip 3 m wide located 3 m from the left sidelines and 3 m from the right sideline of the field and behind the bottom line; zone 5, defined as the strip 3 m wide from the left sideline and behind the bottom line (Queiroga et al., 2010).

Serve type, defined as the type of serve used by the player, considering the location of the player at the time of contact with the ball (García-de-Alcaraz et al., 2016). The categories were: standing, when the player making the throw has some contact with the ground at the moment of hitting the ball; jump, when the player who performs the serve does not have any contact with the ground at the moment of hitting the ball (Afonso et al., 2012; Costa et al., 2012).

Striking technique, the type of serve technique used by the player, considering the flight trajectory of the ball after striking. The categories were: power, when the player who makes the serve contacts by transmits great power and speed to the ball, so the ball rotates forwards; float, when the player who makes the serve contacts the ball with the minimum surface and the least possible time, so the ball does not rotate, but follows a fluctuating and unpredictable trajectory (García-de-Alcaraz et al., 2016).

Serve direction, defined as the direction determined by the serve depending on the serve zone and reception zone. The categories were: parallel, the area of origin of the serve and the reception zone are in line; this direction corresponds to the serves of one-to-five, six-to-six, and five-to-one; mid cross-court, the area of origin of the serve is in an area close to the reception zone; this direction corresponds to the serves of one-to-six, six-to-five, six-to-one, and five-to-six; long cross-court, the area of origin of the serve is in an area far from the reception zone; this direction corresponds to the serves of one-to-one and five-to-five (Gil et al., 2011).

Serve efficacy, defined as the performance or effect obtained with the serve. In order to assess efficacy, the systems of categories employed in “Data Volley System Valuation” (Data Volley, 2010) were used: perfect serve (#), the opponent does not touch the ball or fails to return it; positive serve (+), the opposite reception ends three or more meters from the net, the setter cannot play the first tempo, or the reception ends 1–2 m from the net, making the setter’s combination difficult; negative serve (−), the opposite reception is perfect, the setter has all the pass options.

Receiver player, defined as the in-game role of the player at whom the serve is aimed for reception. The categories were: outside-hitter, the receiver player has an offensive role and is positioned in the attack zone of the court, i.e., zones two, three, and four; receiver of the defence zone, the receiver player has a defensive role and is positioned in the defence zone of the court, i.e., zones one, six, and five; libero, the receiver player is a specialist in defence and reception; other players, the receiver player has a different role from the outside-hitter, receiver of the defence zone, and libero (Gil-Arias et al., 2016).

Reception zone, defined as zone where the serve is received. The categories were: lane one, zone of 3 m × 9 m located on the right side of the court; lane six, zone of 3 m × 9 m located at the centre of the court; lane five, zone of 3 m × 9 m located on the left side of the court; space between players, reception is performed in an intermediate zone with conflict between two receivers (Gil-Arias et al., 2016).

Reception efficacy, defined as the effect obtained in the reception of the serve. In order to assess efficacy, the systems of categories employed in “Data Volley System Valuation” (Data Volley, 2010) were used: perfect serve-receptions (#), defined as the reception through which the ball reaches an optimal setting zone, allowing the pass in suspension and giving the setter all the attack options; positive serve-receptions (+), defined as the reception that gives the setter all the attack options, but in order to play the first tempo, he must take risks; negative serve-receptions (−), defined as the reception that does not give the setter all the attack options, so he cannot make first-tempo attacks.

**FIGURE 1:**
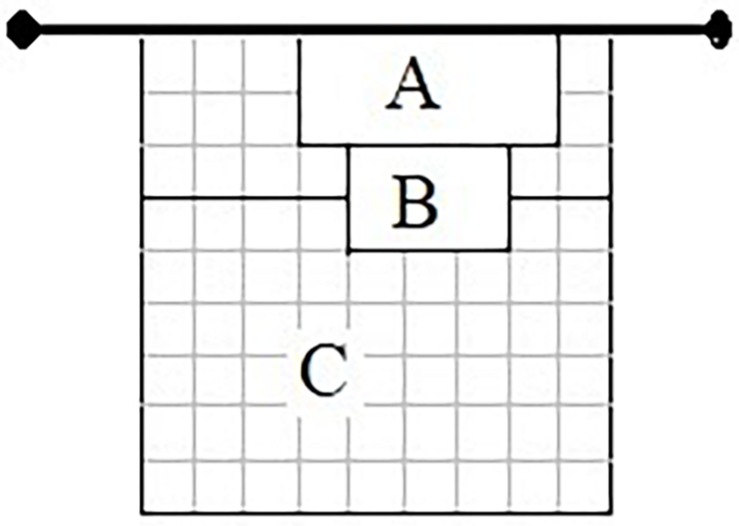
Setting zone (Adapted from Castro and Mesquita, 2010, p. 200).

Setting zone (Figure 1), defined as the place on the court from which the setting pass was carried out. The categories were: an excellent zone, a 10 m^2^ area (2 m × 5 m), located 1 m from the right sideline and 3 m from the left sideline; an acceptable zone, a 6 m^2^ area (2 m × 3 m) located at 2 m from the zone A, at 4 m from the left lateral line and at 2 m from the right lateral line; an non-acceptable zone, all the remaining area (Afonso et al., 2012).

Type of set, defined as the typology of sets made by players, based on whether the setter was in contact with the ground at the time of performing the set. The categories were: jump set, when the setter has his feet in the air at the moment of contact with the ball; standing set, when the setter has his feet on the ground at the moment of contact with the ball (Palao and Martínez, 2013; Palao and Ahrabi-Fard, 2014).

Setting technique, defined as the complete gesture used in the setting pass. The categories were: forearm set, the setter performs the setting touching with the forearm; overhand set, the setter performs the setting touching with the fingers of both hands (Papadimitriou et al., 2004).

Setting efficacy, defined as the performance or effect obtained in the setting. The FIVB system criteria were used. The categories were: non-precise setting, defined as setting that does not allow an attack or that allows an attack but without all the options; good setting, defined as a precise set action that allows an attack in front of two blockers or one middle blocker; and perfect setting, defined as a precise set action that allows an attack in front of one or no blockers (adapted of Moreno et al., 2008).

Set’s area, defined as the area of the court where the attack strike was made. The categories were: zone one, defined as an area 3 m × 6 m long, located in the right area of the rear part of the field; zone two, an area 3 m × 3 m long, located on the upper right side of the network; zone three, an area 3 m × 3 m long, located in the central area of the front of the network; zone four, an area 3 m × 3x long located on the upper left side of the network; zone six, an area 3 m × 6m, located in the central area of the rear of the field (Tsavdaroglou et al., 2018).

Set tempo, defined as the interaction between the moment when the setter makes contact with the ball and the start of the attackers’ approach. The categories were: first tempo, the attacker is in the air when the ball reaches the setter; second tempo, the attacker is performing the penultimate step of the race when the ball reaches the setter; third tempo, the attacker has not started the attack race when the ball reaches the setter (Costa et al., 2011; González-Silva et al., 2016).

### Statistical Analysis

An inferential analysis was performed to verify the associations between each of the criteria and setting efficacy. This analysis is presented through contingency tables, including chi-square and Cramer’s V-values. The statistical significance level considered was *p* < 0.05. The inferential analysis indicated a significant relationship between setting efficacy and: reception efficacy, setting zone, type of set, set’s area, and set tempo. The remaining criteria (in-game role of the server, serve zone, serve type, striking technique, serve direction, receiver player, reception zone, and setting technique) could not be included in the model because they did not show an association. Finally, using the multinomial logistic regression model, the predictions of the criteria on setting efficacy were obtained. We performed a multicollinearity test prior to regression analysis to avoid including intercorrelated criteria. We considered the value of tolerance > 0.10 and FIV < 10 (Hair et al., 2014). As a result of this analysis, serve efficacy (tolerance = 0.031, FIV = 32.253) was excluded as a criterion because it had a value of tolerance below 0.10 and a value of FIV above 10 (Hair et al., 2014). All statistical analyses were performed using the statistical software package SPSS (version.0 for Windows, SPSS, Inc., Chicago, IL, EUA).

## Results

Table 3 presents the results of the TG. The model [P] [V]/[C] was created where the relative and absolute coefficients of the facet, category, in this case, were 0.082. With a generalisation coefficient close to 0, this indicates the heterogeneity of the system and the integrity and mutual exclusivity (E/ME), therefore being the optimal estimated value (Blanco-Villaseñor et al., 2014) so the category system is valid (Table 3).

**TABLE 3:** Generalisability analysis partial models’ adjustments of categories with SAGT.

**Sources of variation**	**Sum of squares**	**Degree of freedom**	**Middle square**	**Random**	**Mixed**	**Corrected**	**%**	**Standard error**
	1640.784	22	74.581	0.129	0.129	0.129	0.212	0.037
[V]	559.801	13	43.062	−1.252	−1.252	−1.252	0.000	0.081
[P][V]	188.332	286	0.659	−0.135	−0.135	−0.135	0.000	0.002
[C]	16490.879	41	402.217	−2.653	−2.653	−2.653	0.000	0.360
[P][C]	4051.253	902	4.491	−0.132	−0.132	−0.132	0.000	0.016
[V][C]	670621.640	533	1258.202	54.429	54.429	54.429	89.372	3.345
[P][V][C]	74387.370	11726	6.344	6.344	6.344	6.344	10.417	0.083

**Design**								
G indices		[P][V]/[C]			[V][C]/[P]			
G relative		0.08			0.99			
G absolute		0.08			0.99			

Table 4 presents the results of the inferential analysis.

**TABLE 4:** Association between criteria and setting efficacy.

**Criteria**	**Chi-cuadrado**	**V de Cramer**	**P**
In game role of the serve	2.038	0.027	0.916
Serve zone	3.324	0.035	0.505
Serve type	1.874	0.037	0.397
Striking technique	0.033	0.005	0.983
Serve direction	3.934	0.038	0.415
Receiver player	3.825	0.037	0.430
Reception zone	9.173	0.058	0.164
Reception efficacy	238.827	0.295	0.000
Setting zone	204.683	0.273	0.000
Type of set	43.748	0.179	0.000
Set’s area	179.759	0.256	0.000
Tempo of set	268.034	0.313	0.000

Table 5 presents the predictive analysis of the reference category perfect setting on the criteria setting efficacy compared to non-precise setting and good setting:

**TABLE 5:** Adjusted model of setting efficacy.

**Criteria**	**Perfect %^a^**	**Non- precise %**	**OR Crude**	**OR Adjusted**	***p***	**Good %**	**OR Crude**	**OR Adjusted**	***p***
**Reception efficacy**									
Positive	36.6	16.5	3.332 (2.194–5.059)^c^	3.328 (1.791–6.184)^c^	**0.000**	46.9	2.014 (1.543–2.629)	1.027 (0.622–1.698)^c^	0.916
Negative	5.8	19.2	24.357 (13.433–44.164)	4.522 (1.667–12.266)	**0.003**	75	20.299 (12.452–33.093)	3.425 (1.511–7.762)	**0.003**
Perfect^b^	⋅	⋅	⋅	⋅	⋅	⋅	⋅	⋅	⋅
**Setting zone**									
Acceptable zone	28.5	13.4	2.555 (1.723–3.789)	0.825 (0.451–1.511)	0.534	58.1	3.156 (2.431–4.098)	2.162 (1.297–3.605)	**0.003**
Non– acceptable zone	5.6	24.1	23.490 (12.145–45.433)	2.614 (0.943–7.245)	0.065	70.3	19.449 (10.790–35.057)	2.694 (1.087–6.678)	**0.032**
Excellent zone^b^	⋅	⋅	⋅	⋅	⋅	⋅	⋅	⋅	⋅
**Type of set**									
Jump set	39	12.7	0.078 (0.031–0.193)	0.361 (0.131–0.998)	**0.049**	48.3	0.120 (0.051–0.279)	0.558 (0.214–1.454)	0.233
Standing set^b^	⋅	⋅	⋅	⋅	⋅	⋅	⋅	⋅	⋅
**Set’s area**									
Zone one	19.9	12.5	1.184 (0.658–2.133)	0.680 (0.352–1.315)	0.252	67.6	1.817 (1.196–2.760)	1.043 (0.644–1.689)	0.864
Zone two	28.7	13.6	0.891 (0.555–1.430)	0.789 (0.470–1.326)	0.371	57.8	1.076 (0.771–1.500)	0.900 (0.618–1.310)	0.582
Zone three	70.1	8	0.216 (0.125–0.373)	0.462 (0.221–0.936)	**0.039**	21.9	0.167 (0.115–0.241)	0.664 (0.385–1.145)	0.141
Zone six	57.7	16.3	0.534 (0.295–0.966)	0.951 (0.485–1.866)	0.884	26	0.240 (0.147–0.392)	0.290 (0.170–0.496)	**0.000**
Zone four^b^	⋅	⋅	⋅	⋅	⋅	⋅	⋅	⋅	⋅
**Tempo of set**									
1 tempo	66.1	10.6	0.107 (0.067–0.171)	0.280 (0.139–0.563)	**0.000**	23.3	0.068 (0.047–0.098)	0.174 (0.100–0.304)	**0.000**
2° tempo	43.1	9.3	0.144 (0.093–0.224)	0.260 (0.156–0.431)	**0.000**	47.6	0.214 (0.156–0.293)	0.473 (0.329–0.682)	**0.000**
3° tempo^b^	⋅	⋅	⋅	⋅	⋅	⋅	⋅	⋅	⋅

*“a” Category of references for the dependent variable. “b” Category of references for the independent variable. “c” Numbers in brackets refer to the 95% confidence interval. Bolded values are indicates “*p*” significance value.*

An inspection of Table 5 shows that, when comparing non-precise and perfect settings, positive and negative serve-receptions led to an increase of non-precise settings instead of perfect settings. When comparing good and perfect settings, negative serve-receptions led to an increase of good setting actions, but to a reduction of perfect setting actions.

Regarding setting criteria, when comparing non-precise and perfect settings, the set’s area and set tempo determined setting efficacy. Specifically, the implementation of jumping rather than supporting, setting toward zone three instead of toward zone four, and sets at first and second tempo instead of at the third tempo decreased the number of non-precise settings.

When comparing good and perfect settings, the setting zone, a set’s area, and set tempo were shown to determine setting efficacy. Specifically, setting from an acceptable or non-acceptable zone rather than from an excellent zone decreased setting efficacy by increasing the amount of non-precise setting. Furthermore, setting toward zone six rather than toward zone four, and making sets at first and second tempo instead of at third times increased setting efficacy by decreasing the number of non-precise settings rather than perfect settings.

## Discussion

The aim objective of this investigation was to establish which criteria of service, serve-receptions, and set determine setting efficacy at the highest level of male volleyball.

First, in order to know the validity of the sample, the TG was applied. The results obtained showed that the criteria used in the category system were valid. The TG has been applied in other works, in different sports, obtaining results similar to those of the present study regarding the validity of the category system (Montoro-Escaño and Hernández-Mendo, 2014; Miranda et al., 2019; Vázquez-Diz et al., 2019).

Of all the initially considered criteria, including those related to serving, serve-reception, and set, only those related to serve-reception and setting determined setting efficacy, that is, only the criteria directly related to the set (previous action and the action itself). None of the serving criteria determined this efficacy. The high and similar level of play across the sample of our study may be one of the reasons why no criteria of this action determined the setting efficacy.

We found that poor serve-receptions influenced subsequent sets. More precisely, we found that poor reception efficacy was associated with a decrease in setting efficacy. There were a greater number of non-precise setting actions following poor receptions. Our results therefore demonstrate that the quality of serve-reception is an important factor for a team’s success (Peña et al., 2013; Paulo et al., 2016). It is necessary to continue in-depth study of the intermediate actions and their relationships with the rest of the game actions. Indeed, reception has been shown to influence both the organisation of the attack, via the set (Joao et al., 2010), and its quality (Afonso et al., 2010). Thus, a negative reception performance can influence the performance of the setter and, consequently, affect the team’s offensive organisation (Bergeles and Nikolaidou, 2011). Therefore, it is necessary for the receiving players to perform specific reception exercises, in order to achieve quality receptions. In this regard, a work in which the same sample was considered that the one analysed in the present study (González-Silva et al., in press) showed that variables such as the previous displacement of the receiver, reception technique and receiver position acted as predictors of reception efficacy. Therefore, these variables could be taken into account in training tasks. Optimum reception efficacy will have a positive influence on the set and, therefore, on the organisation of the attack.

With regard to the setting zone, our results showed that setting efficacy was reduced, with more good settings than perfect settings after sets from acceptable and non-acceptable zones. These results are in line with those found by Afonso et al. (2010), where, in the case of sets from a non-acceptable zone, the subsequent sets were not perfect. Setting zone is related to setting efficacy (Silva et al., 2013) and determines the efficacy and timing of an attack (Afonso et al., 2010). Studies such as that of Silva et al. (2016) show that the sixth rotation discriminates victory. In this rotation, the setter is located near the ideal setting position where it will be possible to perform quickly, thus increasing setting efficacy (González-Silva et al., 2016). These results show the need to reproduce “out of the system” in the training situations so that the setting efficacy is not influenced by the area of arrival of the ball.

The type of set also determined setting efficacy: prior jumping sets increased setting efficacy, decreasing the number of good setting actions relative to perfect setting actions. These results are consistent with those of Palao and Martínez (2013), who showed that the use of jump sets by teams of an international level produced an increase in setting efficacy. The higher level of play in these categories makes the use of jump sets common (Palao and Ahrabi-Fard, 2014). With this action, the setters try to deceive their opponents (Mesquita and Graça, 2002) by reducing the cues that the setter gives to the rivals, reducing the flight time of the ball (Buscà and Febrer, 2012) and providing better conditions for the attack (Palao and Ahrabi-Fard, 2014). This implies that jump sets increase the speed of the game, as well as the attack efficacy, and the efficacy of the action (Palao and Echeverría, 2008). Finally, concerning the set’s area and the set tempo, perfect settings were associated with a faster tempo and more balls being sent to zones three and pipe, compared to non-precise and good setting. Attacks in these zones and at fast times increase the options of obtaining points and limit the defence (Castro et al., 2011). It is therefore advisable that, in training, setters acquire the ability to play quickly as well as with variability concerning the set’s area (Ramos et al., 2017). Therefore, whenever conditions permit, it is preferable to perform jump sets, which increase the speed of play. Likewise, it is advisable to make the settings quickly, as the increase in the speed of the game will allow the attackers to carry out their attacks against a smaller number of rival players in the blockade, thereby favoring gaining a point.

As for the limitations of the study, despite the fact that the study was limited to the 12 best teams in the championship, the quality of the opponent was not taken into account. In the future, these criteria should be considered, as well as other contextual criteria, which provide detailed information of the game context. In addition, in future research, we intend to investigate new statistical tests such as the analysis of social networks.

## Conclusion

At the highest level of male volleyball, considering criteria of actions prior to the set (serve and reception) and of the set itself, only criteria related to reception (reception efficacy) and set (setting zone, type of set, a set’s area, and set tempo) determined setting efficacy. None of the service-related criteria were found to be determinants.

The continuous search for improvement in all game actions at this level of volleyball means that the differences between some teams may be minute. Consequently, the appraisal of the criteria that affect the performance of actions is of vital importance to teams. To improve the performance of the setting action, coaches should consider the influence of criteria of preceding actions (reception efficacy) in addition to criteria related to space, speed, and setting technique. Moreover, it would be advisable not to train only “in the system” but also situations “out of the system” with the aim of achieving some independence between actions, that is, the setter manages to perform assignments efficiently and in specific areas, independently of the conditions in which the ball arrives. Specifically, receivers need to increase reception efficacy in order to ensure that a greater number of balls reach the ideal sets area and to avoid negatively affecting the set. Our results also suggest that setters should make as many jumps sets as possible and be variable concerning the set’s area and the set tempo, preferably making a quick play.

## Data Availability Statement

All datasets generated for this study are included in the article/supplementary material.

## Ethics Statement

The study is exempt from ethical approval because the observation of the game actions does not pose any risk to the participants. It was found to be in accordance with Spanish and international guidelines for scientific research involving humans.

## Author Contributions

JG-S and MM designed the study. JG-S wrote the original manuscript. All authors critically reviewed and revised the draft., read and approved the final version of the manuscript, and agree with the order of presentation of the authors.

## Conflict of Interest

The authors declare that the research was conducted in the absence of any commercial or financial relationships that could be construed as a potential conflict of interest.
